# Structural basis for exploring the allosteric inhibition of human kidney type glutaminase

**DOI:** 10.18632/oncotarget.10791

**Published:** 2016-07-22

**Authors:** Sarath Ramachandran, Catherine Qiurong Pan, Sarah C. Zimmermann, Bridget Duvall, Takashi Tsukamoto, Boon Chuan Low, J. Sivaraman

**Affiliations:** ^1^ Department of Biological Sciences, National University of Singapore, 117543, Singapore; ^2^ Mechanobiology Institute Singapore, National University of Singapore, 117411, Singapore; ^3^ Department of Neurology and Johns Hopkins Drug Discovery Program, Johns Hopkins University, Baltimore, Maryland 21205, USA

**Keywords:** glutaminase, cancer target, BPTES, CB-839, allosteric inhibitors

## Abstract

Cancer cells employ glutaminolysis to provide a source of intermediates for their upregulated biosynthetic needs. Glutaminase, which catalyzes the conversion of glutamine to glutamate, is gaining increasing attention as a potential drug target. Small-molecule inhibitors such as BPTES and CB-839, which target the allosteric site of glutaminase with high specificity, demonstrate immense promise as anti-tumor drugs. Here, we report the study of a new BPTES analog, N, N′-(5,5′-(*trans*-cyclohexane-1,3-diyl)bis(1,3,4-tiadiazole-5,2-diyl))bis(2-phenylacetamide) (trans-CBTBP), and compared its inhibitory effect against that of CB-839 and BPTES. We show that CB-839 has a 30- and 50-fold lower IC_50_ than trans-CBTBP and BPTES, respectively. To explore the structural basis for the differences in their inhibitory efficacy, we solved the complex structures of cKGA with 1*S*, 3*S*-CBTBP and CB-839. We found that CB-839 produces a greater degree of interaction with cKGA than 1*S*, 3*S*-CBTBP or BPTES. The results of this study will facilitate the rational design of new KGA inhibitors to better treat glutamine-addicted cancers.

## INTRODUCTION

One of the hallmarks of cancer cells is their increased reliance on glycolysis for their bioenergetic and biosynthetic requirements, even in the presence of oxygen [1, 2]. In addition to maintaining aerobic glycolysis, cancer cells display a characteristic reprogramming of metabolic pathways and glutamine-dependent anaplerosis to maintain the high flux of intermediates required for their upregulated nucleotide, protein and fatty-acid biosynthesis [3]. Glutaminase is a mitochondrial enzyme that catalyzes the first step in glutamine-dependent anaplerosis by converting glutamine to glutamate, more simply referred to as glutaminolysis. *GLS1* and *GLS2* encode two primary isoforms of human glutaminase. *GLS1* has two full-length splice variants, kidney-type glutaminase (KGA) and glutaminase-C (GAC), whereas *GLS2* encodes liver-type glutaminase (LGA) [4]. Both GLS1 isoforms share an identical catalytic domain.

Glutaminase is also reportedly upregulated in response to oncogenes such as c-Myc, Raf, Ras and the Rho GTPase [5–8] and, because of this, glutaminase is an emerging target for cancer therapeutics [7, 9–11]. Indeed, treating tumor cells with an antisense glutaminase mRNA induces apoptosis under oxidative stress [12]. To date, several approaches using small molecules have been used to inhibit glutaminase [8, 13]. Competitive inhibitors, such as 6-diazo-5-oxo-L-norleucine (DON) and azaserine, have had limited success against tumors owing to their severe toxicity and non-specificity [13–15]. Two classes of allosteric inhibitors have been reported, and these vary in their inhibition mechanism: (1) compound-968 (5-(3-bromo-4-(dimethylamino)phenyl)-2,2-dimethyl-2,3,5,6 tetrahydrobenzo[*a*]phenanthridin-4(1*H*)-one) and (2) BPTES (bis-2-(5-phenylacetamido-1,3,4-thiadiazol-2-yl)ethyl sulfide). Compound-968 is specific to *GLS* but shows limited potency in the presence of the inorganic phosphates that promote *GLS* activation by tetramerization [7]. BPTES, on the other hand, is a *GLS1*-specific inhibitor that is active even in the presence of inorganic phosphates [16], with some anti-tumoral success against breast, glioblastoma and B lymphoma cancer cell lines [10, 17–19]. A recent study suggests that the concomitant use of glutaminolysis inhibitors and antagonists against certain tumor-derived chemokines, such as IL-8 [20], could improve the antitumor effect.

In the past, we have demonstrated the inhibition mechanisms for an active site inhibitor, DON, and an allosteric inhibitor, BPTES [8, 13]. Others and we have reported that BPTES inhibits KGA by binding to an allosteric site between a pair of homodimers, and this results in a dramatic conformational change in key loop residues (Glu312-Pro329) that render the tetramer inactive [8, 21]. Although, BPTES has shown promise in the treatment of a range of cancer types, its low solubility and moderate potency has limited its pharmacological applications [22]. In an attempt to circumvent the potency and solubility limitations of BPTES, various BPTES analogs have been developed. One promising candidate, CB-839, first characterized by Gross *et al.*, is currently undergoing clinical trials. Compared with BPTES, CB-839 is reported to have higher solubility and superior antiproliferative activity in triple-negative breast cancer cells [19]. Furthermore, CB-839 has a 13-fold lower IC_50_ value, slower reversibility kinetics, and, most importantly, a stronger affinity for *GLS1* over BPTES [23]. The compound also demonstrates antitumor efficacy in acute myeloid leukemia (AML), multiple myeloma, solid tumors and hematological malignancies [24–26]. Despite its current successes, the structural basis for this potent inhibitory activity of CB-839 has not been determined.

As a part of our continued efforts to understand the allosteric inhibitory mechanism of KGA and develop superior inhibitors, here we characterized the inhibitory efficacy and toxicity of a new BPTES analog developed by Agios (Cambridge, MA), N,N′-(5,5′-(*trans*-cyclohexane-1,3-diyl)bis(1,3,4-tiadiazole-5,2-diyl))bis(2-phenylacetamide), hereafter referred to as trans-CBTBP [27], and compared it against CB-839 and BPTES. Additionally, we determined the crystal structures of the cKGA (catalytic domain Ile221-Leu533 of KGA) in complex with the trans-CBTBP enantiomer (1*S*, 3*S*-CBTBP), and CB-839. These studies have enhanced our understanding of how specific modifications can affect the inhibitory potency of the compound and will aid in the design of new lead molecules for therapeutics.

## RESULTS

### Exploring the allosteric interactions involved in the inhibition of KGA

**Figure 1:**
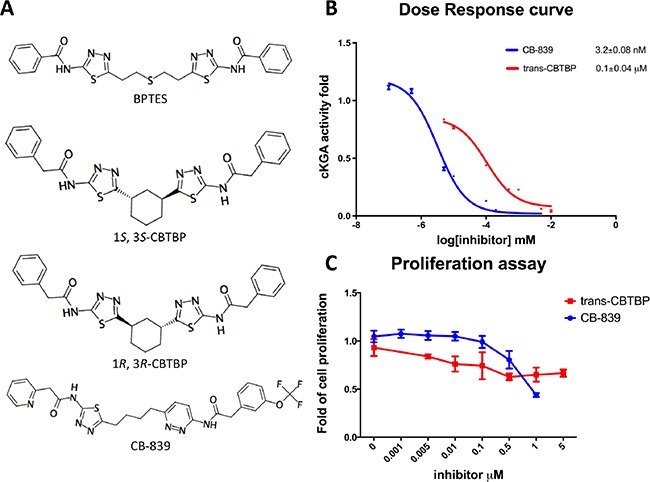
Inhibition assay for cKGA with trans-CBTBP and CB-839 **A.** Chemical structures for BPTES, trans-CBTBP and CB-839. Trans-CBTBP used for the studies is a racemic mixture of the 1*S*, 3*S* and 1*R*, 3*R* enantiomers. **B.** Dose response curves for trans-CBTBP and CB-839. **C.** Cell viability assay for trans-CBTBP and CB-839.

Our previous structural studies on the allosteric inhibition of cKGA with BPTES reported two primary interactions between cKGA and BPTES: (1) Hydrogen bonding contacts between cKGA and the thiadiazole moiety and the amide nitrogen of BPTES; (2) Hydrophobic interactions between the cKGA allosteric pocket and aliphatic BPTES linker [8]. In an attempt to explore the role of these interactions in terms of their effect on inhibitor affinity, we chose two representative BPTES analogs, trans-CBTBP and CB-839 to determine the mode of cKGA inhibition. Trans-CBTBP is structurally similar to BPTES, with the exception of a 1,3-di-substituted cyclohexyl ring in place of the acyclic aliphatic linker on BPTES. CB-839, unlike BPTES and trans-CBTBP, is asymmetric across its center and has multiple unique moieties, including the replacement of the terminal phenylacetyl groups with a pyridylacetyl group and a trifluoromethoxylphenyl acetyl group, as well as the substitution of a pyridazine ring for one of the two thiadiazole rings (Figure 1A).

### Inhibition of cKGA by trans-CBTBP and CB-839

In order to compare the inhibitory efficacies of the three inhibitors: BPTES, trans-CBTBP and CB-839, we performed both *in-vitro* and *in-vivo* inhibition assay with recombinant cKGA (Figure 1B; Supplementary Figure S3). Among the three inhibitors, CB-839 showed the lowest IC_50_ values. Although the *in-vitro* assay shows that both BPTES and trans-CBTBP demonstrate similar IC_50_ values; in comparison with BPTES, trans-CBTBP displays a smaller number of rotatable bonds (NRB). The reduction in NRBs (8 in trans-CBTBP vs. 12 in BPTES) in trans-CBTBP would improve the probability of good absorption [28].

Figure 1B shows the dose-response curves for these two inhibitors in 293T epithelial cells. The IC_50_ values for glutaminase inhibition for trans-CBTBP and CB-839 were determined to be 0.1 μM and 3.2 × 10^−3^ μM, respectively, with a 30-fold difference in activity between the two compounds. Further, we note that the IC_50_ for trans-CBTBP is only a moderate improvement over that for BPTES (IC_50_= 0.16 μM) [8] and could be due to greater cell permeability of trans-CPTBP over BPTES. To confirm that glutaminase activities measured were attributed from ectopic cKGA, glutaminase activity were assessed in the absence of ectopic KGA. The endogenous glutaminase activity was 3% of the ectopic expressed cKGA (Supplementary Figure S4). In addition, the endogenous glutaminase was completely inhibited at very low concentrations (1nM) of inhibitors.

Next, to verify the toxicity of the inhibitors to non-tumorigenic cells, we conducted a cell viability assay (Figure 1C). We found that neither CB-839 nor trans-CBTBP caused substantial cytotoxicity at concentrations up to 0.1 μM, suggesting that both inhibitors are safe at their effective concentration range.

Overall, we find CB-839 to be a more potent inhibitor than trans-CBTBP and BPTES, with trans-CBTBP showing only a small improvement in its in vivo inhibitory activity over BPTES. To further understand these differences in potency, we solved the complex crystal structures of both inhibitors with cKGA.

### cKGA: *1S,3S*-CBTBP Complex

**Figure 2:**
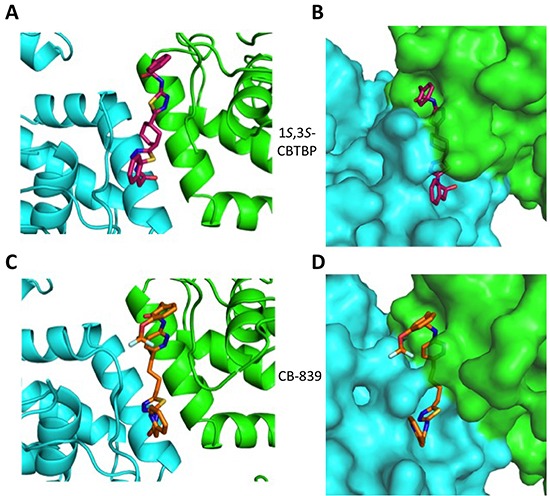
Structure of cKGA in complex with 1S, 3S-CBTBP and CB-839 Close-up view of the inhibitor binding pocket at the dimer interface of cKGA for the **A.** cKGA:1*S*, 3*S*-CBTBP complex and the **C.** cKGA:CB839 complexes. **B.** Molecular surface representation of the inhibitor binding pocket for cKGA and stick representation for 1*S*, 3*S*-CBTBP. **D.** Molecular surface representation of the inhibitor binding pocket for cKGA and CB-839. Structure related figures of this manuscript were generated using PyMOL [50].

A racemic mixture of trans-CBTBP enantiomers: 1*S*, 3*S* and 1*R*, 3*R* were used for co-crystallization with cKGA. The cKGA: 1*S*, 3*S*-CBTBP was found to crystallize and the structure was determined at 2.74 Å resolution (Table 1; Figure 2A, 2B). However multiple crystallization conditions did not yield the crystals of cKGA: 1*R*, 3*R*-CBTBP complex. This indicated a higher preference of the 1*S*, 3*S* stereoisomer for cKGA over the 1*R*, 3*R* form. The 1*S*, 3*S*-CBTBP molecule was positioned using a *F_o_*-F*_c_* Simulated Annealing omit map (Supplementary Figure S1A).

**Table 1:** Data collection and refinement statistics for cKGA:1*S*, 3*S*-CBTBP and cKGA:CB-839 complexes

	cKGA: 1*S*, 3*S*-CBTBP complex	cKGA: CB-839 complex
Space group	I4_1_22	P1
Cell parameters (Å, °)	a= b= 139.57,c= 156.61, α= β=γ=90	a= 126.40,b= 126.63,c= 126.27, α=112.88, β=102.81,γ=112.74
Resolution range (Å)	30-2.74 (2.79-2.74)	30-2.1 (2.14-2.10)
Wavelength (Å)	1.000	1.000
Observed *hkl*	144179	1265236
Unique *hkl*	20506	337350
Completeness (%)	99.5(94.9)	96.60(93.9)
Overall *I/*σ*I*	20.27(1.75)	15.65(2.03)
^a^R_sym_	0.093 (0.572)	0.108 (0.448)
	***Refinement and quality of the model***	
*Resolution range	29.29-2.74	21.4-2.1
^b^R_work_ (%) no. reflections	20.86 (20494)	18.43 (337261)
^c^R_free_ (%) no. reflections	24.86 (1905)	20.50 (1981)
	Root mean square deviation	
Bond length (Å)	0.009	0.009
Bond angle (°)	1.17	1.08
	Ramachandran plot (%)	
Favored region	92.0	98.0
Allowed regions	7.7	2.0
Disallowed regions	0.3	0
	^d^Average B-factors (Å^2^)	
Overall average	65.90	48.40
Macromolecules	65.70	48.00
Ligand	78.80	61.10

a R_sym_ = |I_i_ − <I>|/|I_i_| where I_i_ is the intensity of the i^th^ measurement, and <I> is the mean intensity for that reflection.

b R_work_ = |F_obs_ − F_calc_|/|F_obs_| where F_calc_ and F_obs_ are the calculated and observed structure factor amplitudes respectively.

c R_free_ is as for R_work_, but only for approx. 9% and 0.6% of the total reflections chosen at random and omitted from refinement for cKGA: 1*S*, 3*S*-CBPTBP and cKGA:CB-839 respectively.

d Individual B-factor refinement was carried out.

**Figure 3:**
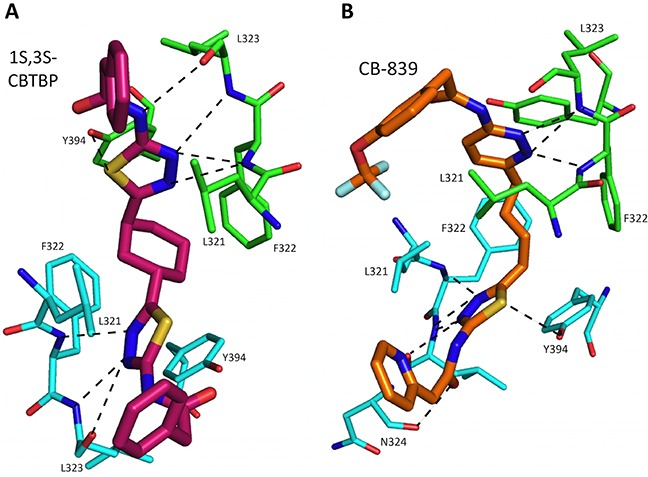
Interactions of 1S, 3S-CBTBP and CB-839 with cKGA **A.** Hydrogen bonding interactions involved in cKGA: 1*S*, 3*S*-CBTBP binding. **B.** Interactions between CB-839 and allosteric site of cKGA. For clarity not all the protein side chains are shown in these figures.

Trans-CBTBP shares an internal symmetry across its 1,3-di-substituted cyclohexyl linker (Figure 1A). BPTES and 1*S*, 3*S*-CBTBP share similar symmetric halves, and have identical hydrogen bonding interactions with cKGA (Figure 3A, Supplementary Table S1). However the differences in the hydrophobic interactions of these two inhibitors with cKGA is prominent. Unlike the aliphatic linker of BPTES, the cyclohexane linker from 1*S*, 3*S*-CBTBP forms multiple hydrophobic interactions with the side chains of Tyr394, Phe322 and Leu321 from both the neighboring cKGA monomers (see Supplementary Table S2).

### cKGA: CB-839 Complex

The complex structure for cKGA:CB-839 was determined at 2.1 Å (Table 1; Figure 2C, 2D). Two molecules of CB-839 are found to be interacting with the cKGA tetramer. Similar to BPTES and 1*S*, 3*S*-CBTBP, several hydrogen-bonding interactions mediate the binding of CB-839 with cKGA (Supplementary Table S1). Similar to that observed for the cKGA:1*S*, 3*S*-CBTBP complex, the cKGA backbone amide groups of Phe322 and Leu323 are involved in hydrogen bonding contact with CB-839. Notable unique interactions include the hydrogen bonds between the pyridazinyl and acetyl groups of the inhibitor with the side-chains of Try394, Lys320 and Asn324 from cKGA. Furthermore, the thiadiazol group of CB-839 is also involved in a water-mediated interaction with Asp327 of cKGA. The terminal moieties of the inhibitor–fluorine and oxygen atoms of the trifluromethoxy phenyl group, as well as the nitrogen atoms from the pyridine ring of the inhibitor are not engaged in any hydrogen bonding contact with cKGA. It is plausible, however, that the trifluoromethoxy phenyl group could help improve the solubility of CB-839 [29, 30].

Hydrophobic interactions between the aliphatic linker of CB-839 and the side-chains of Phe322 and Tyr394 from cKGA also contribute to the binding (Supplementary Table S2). The pyridinyl moiety and terminal phenyl ring also interact with the hydrophobic side-chain carbon atoms of Glu325 and Leu321, respectively.

### Comparison of the binding of BPTES, *1S, 3S*-CBTBP and CB-839 with cKGA

**Figure 4:**
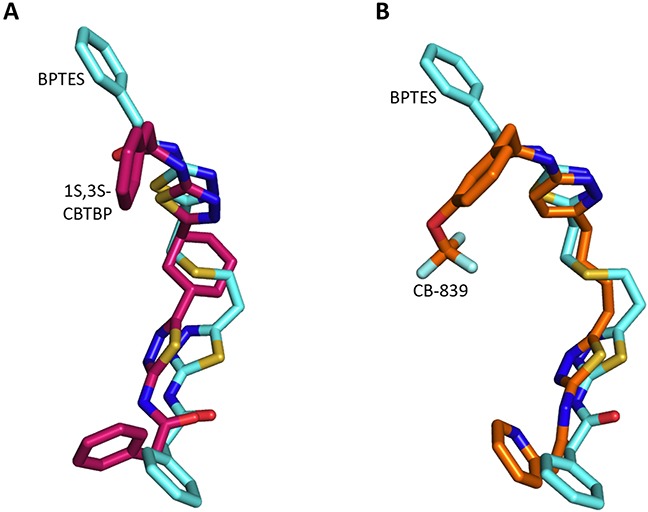
Superposition of inhibitors **A.** Comparison of 1*S*, 3*S*-CBTBP with BPTES. **B.** Comparison of CB-839 with BPTES.

A comparison of the cKGA complex structures of BPTES, 1*S*, 3*S*-CBTBP, and CB-839 indicates that binding differences are primarily due to the strengths of interactions made with the allosteric pocket of cKGA. The cKGA allosteric pocket resembles a lock, with negligible changes observed in its conformation in complex with the different inhibitors; the inhibitors are analogous to different keys with their moieties acting as levers. The binding affinity is a function of the flexibility and orientation of the interacting moieties from the inhibitors (Figure 4).

The interaction of 1*S*, 3*S*-CBTBP with the cKGA allosteric pocket is primarily mediated by hydrogen bonding interactions from the thiadiazol group and hydrophobic interactions from the cyclohexane linker. In comparison, the driving force for CB-839 binding is the higher number of hydrogen bonds and this is assisted by the hydrophobic interactions from the pyridinyl ring. The lower effective concentrations for CB-839 are consistent with an improved cumulative strength of the interactions. The calculated buried surface areas of 520 Å^2^ for CB-839 and 460 Å^2^ for 1*S*, 3*S*-CBTBP further validate the greater relative strength of the CB-839 interaction [31].

## DISCUSSION

Metabolic pathway enzymes are increasingly being investigated as potential drug targets for cancer treatment [32–34]. The mitochondrial enzyme glutaminase in particular is under intense investigation in many tumor models [35], with an immense amount of time invested in the development of selective and non-toxic small-molecule glutaminase inhibitors with superior metabolic stability and pharmacodynamics. Various recent efforts have shown that inhibition of glutaminolysis offers synergistic effects when administered alongside anti-NOTCH1, β-lapachone, or BCL-2 inhibition for the treatment of T cell acute lymphoblastic leukaemia (T-ALL), pancreatic cancer, or acute myeloid leukemia (AML), respectively [36–38]. A structural understanding of the differences in the protein: inhibitor interactions as a result of the small modifications to these inhibitors will help in the development and rational design of more potent inhibitors.

In this study, we report the inhibitory efficacies along with complex structural studies of two analogs of BPTES, trans-CBTBP and CB-839. As shown in Figure 1B, we observe a 50-fold lower IC_50_ value for CB-839 as compared with BPTES (30-fold as compared with trans-CBTBP), when using a cell-culture based assay to determine the dose response. This is consistent with the inhibition assay performed by Gross *et al.* for GAC with CB-839 [23]. Further, we have explored the utility of the inhibitors as drugs by correlating their effective concentration range to their toxicities. Both trans-CBTBP and CB-839 were found to be safe at their IC_50_ concentrations. Intriguingly, increasing CB-839 inhibitor treatment starting from 0.5mM, leads to rapid decrease of proliferation. In contrast, trans-CBTBP treatment does not have a major impact on the proliferation of cells, suggesting lower toxicity of trans-CBTBP over CB-839 (Figure 1C). The mechanism of cell death at higher concentrations of CB-839 is yet to be determined and warrants further investigation.

Both 1*S*, 3*S*-CBTBP and CB-839 were found to interact with the same allosteric pocket of cKGA, as reported for BPTES. The effective concentrations of trans-CBTBP and CB-839 were compared with BPTES and its other analogs (Table 2). The low affinity of inhibitors 4 and 3 for cKGA could be attributed to the absence of the terminal phenyl ring and acetyl group, which are involved in interactions with the cKGA allosteric pocket as observed in the crystal structure of the cKGA:BPTES complex. Inhibitor 2 likely has better binding because of the central sulfur atom, which restrains the flexibility of the diethyl sulfide linker. The terminal phenyl rings on inhibitor 5 that are substituted with electron-donating methoxy groups, leads to moderate improvements in its affinity over inhibitors 2, 3 and 4. Unlike BPTES, the cyclohexane linker of 1*S*, 3*S*-CBTBP improves the hydrophobic interactions and imposes rigidity across the center of the inhibitor. The terminal electron-withdrawing trifluoromethoxy group of CB-839, substituted on the phenyl ring on BPTES, deactivates the aromatic ring system and increases the electronegativity of the pyridazinyl nitrogen atoms, thus strengthening the hydrogen bonding interactions. In addition the trifluoromethoxy group increases the lipophilicity of the inhibitor.

**Table 2:** A comparison of KGA allosteric inhibitors

Inhibitor Structure	IUPAC name	IC_50_ μM	Reference
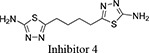	5-(4-(5-amino-1,3, 4-thiadiazol-2-yl)butyl)-1,3,4-thiadiazol-2-amine	18.60	[7]
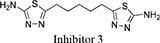	5-(5-(5-amino-1,3,4-thiadiazol-2-yl)pentyl)-1,3,4-thiadiazol-2-amine	11.10	[7]
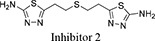	5-(2 -((2-(5-amino-1,3,4-thiadiazol-2-yl)ethyl)sulfanyl)ethyl)-1,3,4-thiadiazol-amine	8.02	[7]
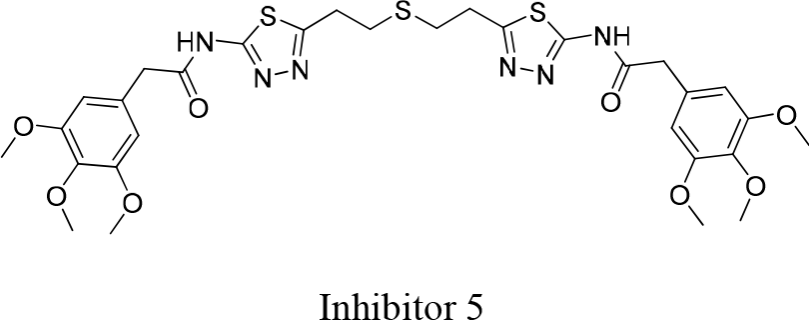	2,3,4-trimethoxy-N-(5-(2-((2-(5-(3,4,5-trimethoxybenzamido)-1,3,4-thiadiazol-2-yl)ethyl)sulfanyl)ethyl)-1,3,4-thiadiazol-2-yl)benzamide	5.44	[7]
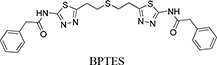	bis-2-(5-phenylacetamido-1,3,4-thiadiazol-2-yl)ethyl sulfide	0.16	[7,20]
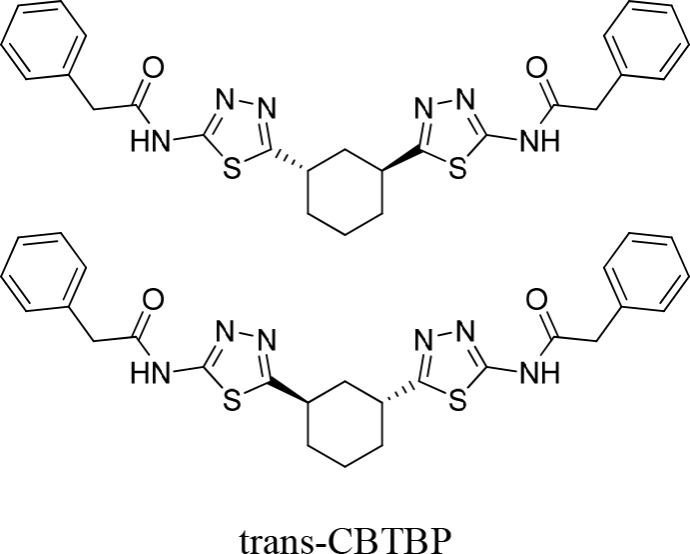	N,N′-(5,5′-((*1S,3S*)-cyclohexane-1,3-diyl)bis(1,3,4-tiadiazole-5,2-diyl))bis(2-phenylacetamide) N,N′-(5,5′-((*1R,3R*)-cyclohexane-1,3-diyl)bis(1,3,4-tiadiazole-5,2-diyl))bis(2-phenylacetamide)	0.10	This study
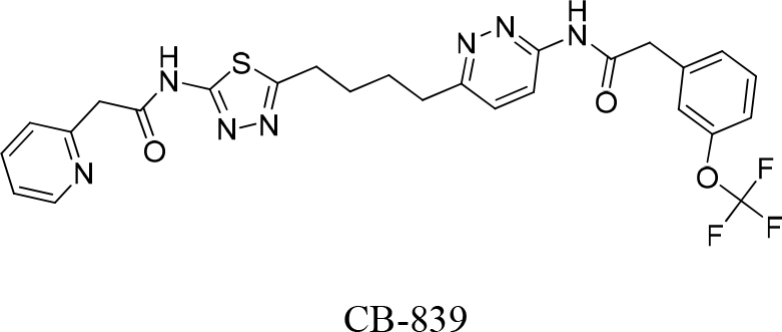	2-(pyridin-2-yl)-N-(5-(4-(6-(2-(3-(trifluoromethoxy)phenyl)acetamido)pyridazin-3-yl)butyl)-1,3,4-thiadiazol-2-yl)acetamide	0.003	This study

The findings from these studies provide further insights into rational design of the next generation of KGA inhibitors by utilizing knowledge garnered from ligand-based interactions. From the activity assay, we have shown that CB-839 has enhanced inhibitory efficacy over trans-CBTBP and BPTES. Since the terminal atoms of the inhibitors do not engage in key interactions with the cKGA, chemical modifications should be targeted around this region to improve the solubility of new inhibitors [39]. Since both GAC and KGA share exactly the same catalytic domain, the structural and inhibition studies performed with cKGA would be similar and applicable to the glutaminase domain of GAC as well. Moreover, building on what we now know, a chimeric inhibitor with a cyclohexane linker and a pyridazinyl ring might improve the interactions of the compound with KGA and GAC [27].

## MATERIALS AND METHODS

### Inhibitor synthesis

For this study, we used a racemic mixture of two trans-CBTBP enantiomers: 1*S*, 3*S* and 1*R*, 3*R*. Between the two enantiomers, only 1*S*, 3*S*-CBTBP was found to crystallize with cKGA. The racemic mixture of trans-CBTBP was prepared as reported previously [27]. For both the inhibition and proliferation assay, the racemic mixture of trans-CBTBP was used. Likewise, CB-839 was prepared by the previously reported method [40].

### Cloning, expression and purification

The cKGA was cloned into pET-28(b) fused to an N-terminal His tag, and expressed in *Escherichia coli* BL21 (DE3)-RIL-Codon plus cells. The cells were induced with 200 μM IPTG and then sonicated in the lysis buffer consisting of 50 mM HEPES pH 7.5, 500 mM NaCl, 10% glycerol, 1 mM DTT, 0.1% (vol/vol) Triton X-100, and one tablet of complete protease inhibitor mixture (Roche Diagnostics, Risch-Rotkreuz, Switzerland). The cell lysate was bound to Ni-NTA affinity beads (Roche Diagnostics) and eluted with 400 mM imidazole. The protein was further purified using a Superdex-200 column (GE Healthcare, Buckinghamshire, UK) in a buffer containing 20 mM HEPES pH 7.5, 200 mM NaCl, 5% glycerol and 3 mM DTT. The protein was then concentrated to 20 mg/ml concentration using protein concentrators (Vivaspin, Sartorius AG, Goettingen, Germany).

### Cell culture, transfection and inhibitors treatment

Human embryonic kidney epithelial 293T cells were maintained in RPMI-1640 (Hyclone, GE Life Sciences) with 10% (vol/vol) FBS (Gibco-BRL, Gaithersburg, MD), 2 mM L-glutamine, 100 U/ml penicillin, 100 μg/ml streptomycin (HyClone). Cells were grown at 37°C in 5% CO_2_. Confluent cells grown in 12-well plates were transfected using TransIT (Mirus Bio, Madison, WI) with 1 μg of plasmid encoding wild-type KGA and then incubated with the indicated inhibitors for 24 h.

### *In-vitro* glutaminase assay

The glutaminase assay was performed in two steps using a dual enzyme assay [41]. Inhibitors were dissolved in DMSO. 10 μl of DMSO/inhibitor was incubated with 80 μl assay mixture A (1 μM recombinant cKGA, 50 mM Tris-acetate (pH 8.6), 100 mM Potassium phosphate, and 0.2 mM EDTA) for 2 h at room temperature (24°C). 10 μl of 200 mM glutamine was then added prior to a 20 min incubation at 37°C. The reaction was quenched with the addition of 10 μl of 0.6 M HCl. The reaction mixture was then incubated for 30 min at room temperature (24°C) with 100 μl of the assay mix B (3.7 U glutamate dehydrogenase, 160 mM Tris-acetate (pH 9.4), 400 mM hydrazine, 5 mM ADP, and 2 mM nicotinamide adenine dinucleotide) and the absorbance read at 340 nm using an Infinite 200 microplate reader (Tecan, Durham, NC).

### Glutaminase assay with 293T cell line

A glutaminase assay was performed using the two-step procedure as described previously [42]. Confluent 293T cells grown in 12-well plate were transfected with cKGA plasmid and incubated with the indicated inhibitors for 24 h. Cells were lysed with 100 μl of Hepes buffer [20 mM Hepes (pH 7.4)], 150 mM NaCl, 1 % NP40, 20 mM glycerol-2-phosphate, 1 mM sodium orthovanadate, and 20mM sodium fluoride and protease inhibitors (Roche Applied Science, Mannheim, Germany). Lysates were quantified for protein amount using BCA protein assay kit (Pierce Biotechnology, Rockford, IL). Cells were incubated with 10 μl cKGA-inhibitor at 37°C for 10 min with 10 μl of assay mix consisting of 20 mM glutamine, 50 mM Tris-acetate (pH 8.6), 100 mM phosphate, and 0.2 mM EDTA. The reaction was quenched with the addition of 2 μl of 3 M HCl. The reaction mixture was then incubated for 30 min at room temperature (24°C) with 200 μl of the second assay mix (2.2 U glutamate dehydrogenase, 80 mM Tris-acetate (pH 9.4), 200 mM hydrazine, 0.25 mM ADP, and 2 mM nicotinamide adenine dinucleotide) and the absorbance read at 340 nm using an Infinite 200 microplate reader (Tecan, Durham, NC).

### Cell proliferation assays

293T cells were trypsinized and replated into 96-well plates and then treated with inhibitors for 48 h. 293T cells treated with DMSO served as the control. The amount of cell proliferation was determined using an MTT proliferation assay kit (Promega, Fitchburg, WI) as described by the manufacturer. At least three independent experiments were carried out, each with multiple replicates. Statistical significance was analyzed using ANOVA and the Student-Newman-Kuels multiple range test (StatsDirect, Cheshire, UK). Data are mean ± SD (*p* < 0.05) and are expressed as fold increase over the control cells.

### Crystallization and structure determination

The protein:inhibitor complex was prepared by incubating cKGA (2 mg/ml) with inhibitors (1:10 molar ratio; final concentration of 5% DMSO) at 4°C for 1 h. The complex was then concentrated to 20 mg/ml before crystallization screening using the vapor diffusion method. Crystals for cKGA–CB839 was obtained with 0.1 M Bis-Tris propane (pH 7), 3% DMSO and 1.8 M LiSO_4_. cKGA–trans-CBTBP crystals were obtained with 0.1 M Bis-Tris propane (pH 7), 4% DMSO and 2 M LiSO_4_. The crystals were cryopreserved in reservoir solution supplemented with 15% glycerol. For both complexes, the diffraction data sets were collected at the synchrotron beamline 13B1 (wavelength 1.000 Å) at the National Synchrotron Radiation Research Centre (NSRRC, Taiwan). Data sets were processed and scaled using HKL2000 [43].

### Structure solution and refinement

Structures of the cKGA:inhibitor complexes were obtained by molecular replacement with the program Phaser-MR [44] using the coordinates of *apo* cKGA as the search model (Protein data bank, 3VOY). The restraints for the inhibitors were generated using eLBOW program [45, 46]. The model was examined and built in COOT [46] and subsequent refinement was carried out with Phenix-refine [47]. The electron density for the cKGA gating loop (^316^LRFNKL^321^) region is disordered in 1*S*, 3*S*-CBTBP and CB-839 inhibitor complex structures, and was not modeled [48]. The final structures for cKGA:1*S*, 3*S*-CBTBP and cKGA:CB-839 were refined up to 2.74 Å and 2.1 Å resolution, respectively (Table 1). The overall geometry of the final model was analyzed using a Ramachandran plot with the program PROCHECK [49].

## SUPPLEMENTARY MATERIALS FIGURES AND TABLES


