# Supplementary material for: Structural basis for exploring the allosteric inhibition of human kidney type glutaminase
Source: Oncotarget. 2016 Jul 22;7(36):57943–54. doi: 10.18632/oncotarget.10791 (PMC5295402; doi:10.18632/oncotarget.10791)
Supplement: Supplementary file 1 [file oncotarget-07-57943-s001.pdf]

Structural basis for exploring the allosteric inhibition of human kidney type glutaminase

SUPPLEMENTARY MATERIALS

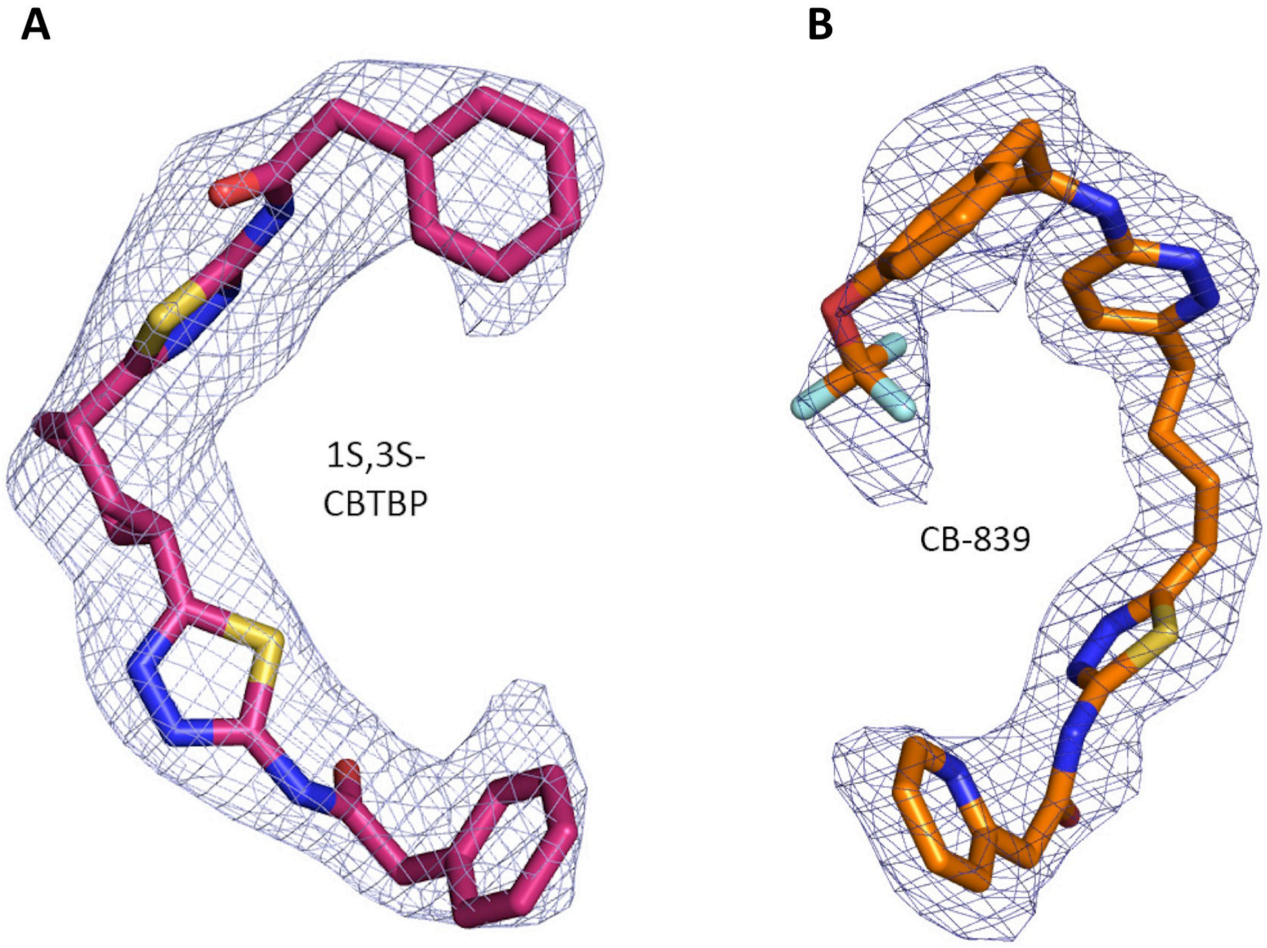

Supplementary Figure S1: Simulated Annealing omit map displaying the electron density for 1*S*, 3*S*-CBTBP and CB-839. The inhibitor and a distance of 3.5 Å surrounding the inhibitors were omitted prior to refinement and map calculation. The map is contoured at 3 σ.

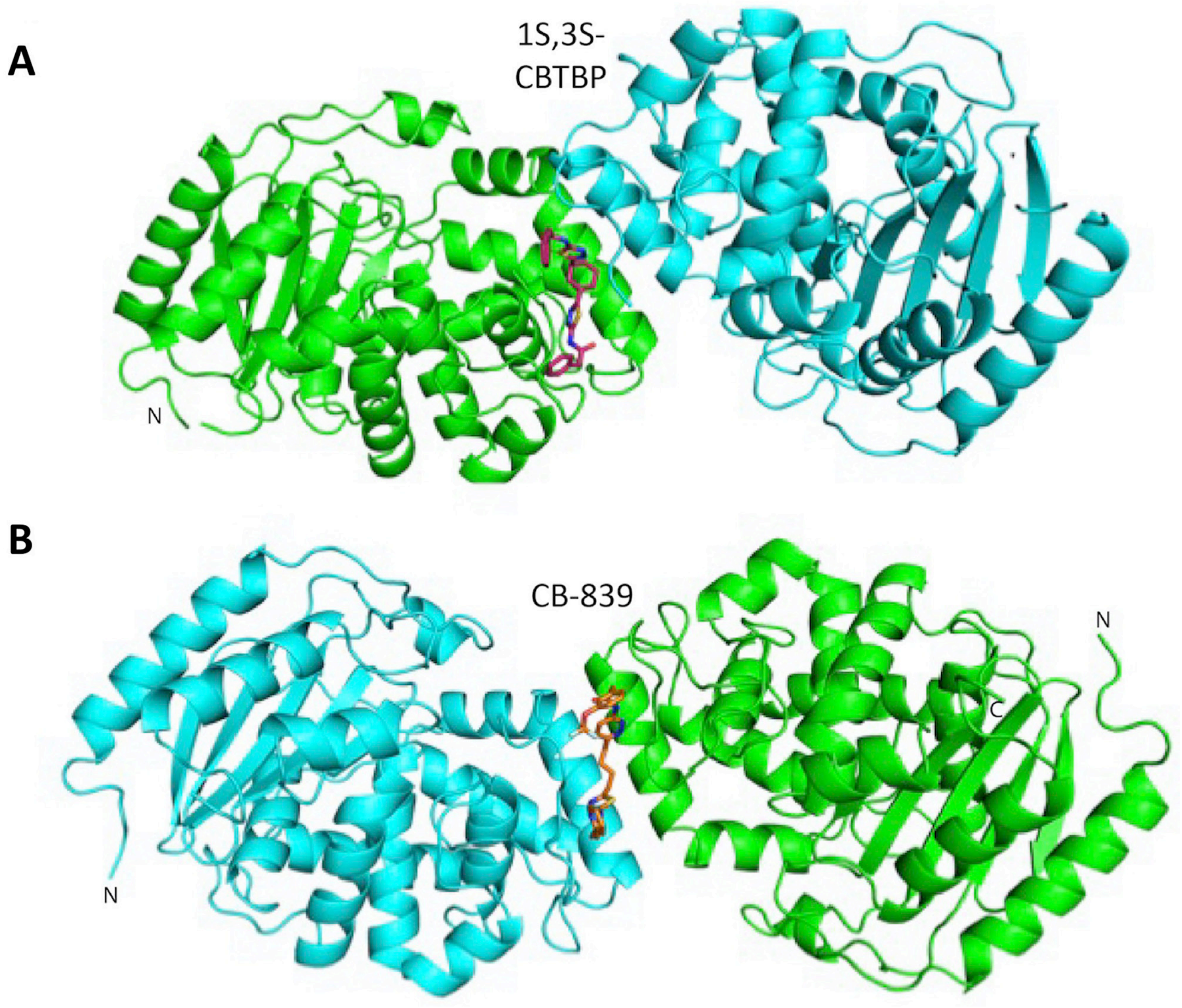

Supplementary Figure S2: Cartoon representation of the cKGA bound with A. 1*S*, 3*S*-CBTBP and B. CB-839 complex. The inhibitors are in stick representation.

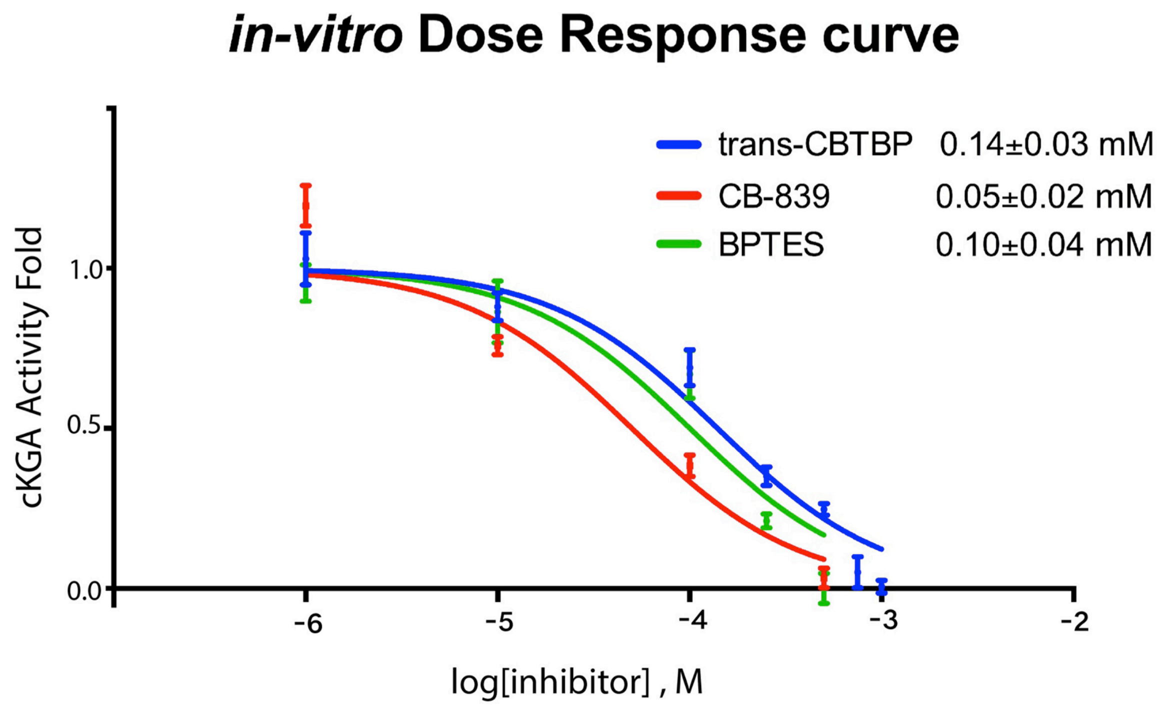

Supplementary Figure S3: *In-vitro* inhibition assay for cKGA inhibitors: BPTES, trans-CBTBP and CB-839.

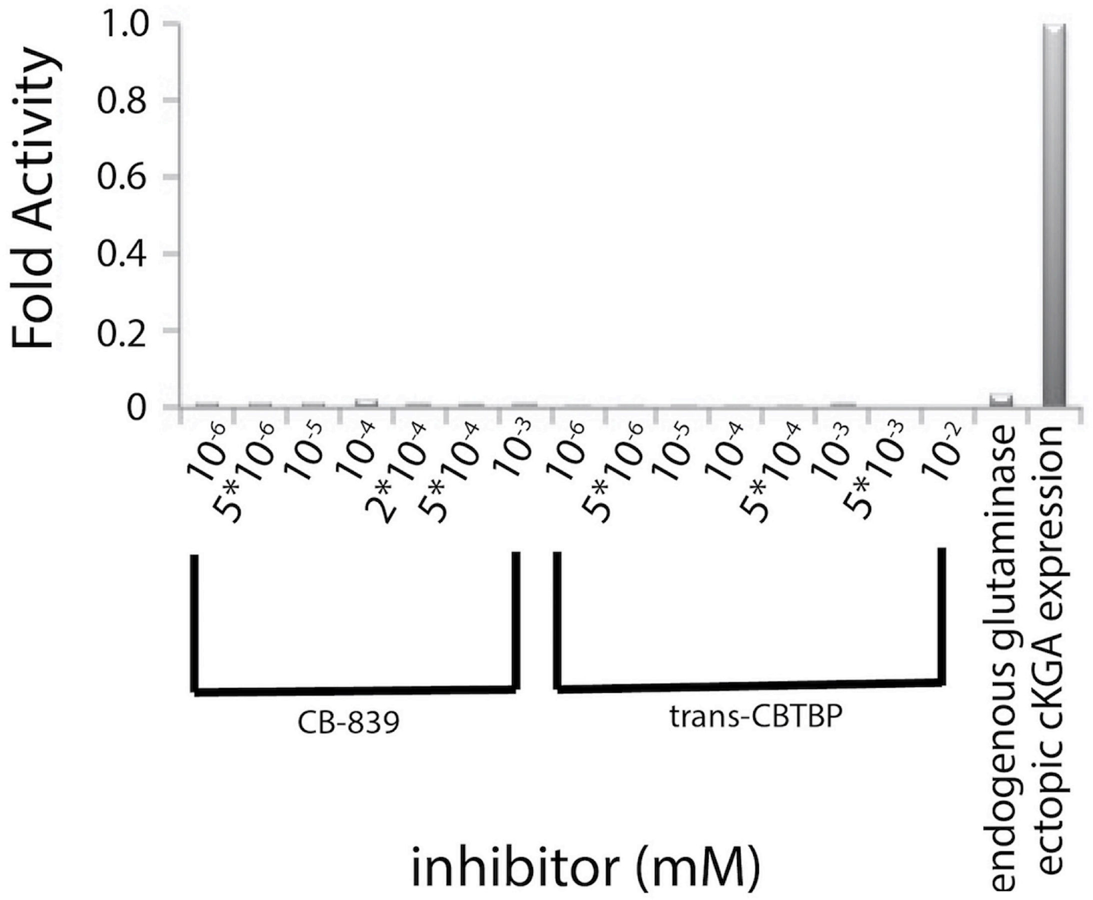

Supplementary Figure S4: Inhibition assay for 293T cells with endogenous glutaminase. Endogenous glutaminase only contributes to about 3% activity of ectopic expressed cKGA.

Supplementary Table S1: Hydrogen bond interactions for cKGA with inhibitors

Inhibitor	Inhibitor atom	Interacting cKGA atom	Distance (Å)
CB-839	Pyridazinyl group:		
	N39	Phe322N	2.9
	N40	Phe322N	3.1
	N40	Leu323N	3.1
	Thiadiazol group:		
	N25	Phe322N	2.9
	N25	Leu323N	3.3
	N26	Leu323N	3.3
	N26	Leu323O	3.2
	S38	Tyr394OH	3.5
	Others:		
	O30	Leu323O	2.9
	O30	Asn324O	3.1
	N15	Leu323O	2.8
1*S*, 3*S*-CBTBP	Thiadiazol group:		
	N02	Phe322N	3.0
	N03	Phe322N	3.0
	N03	Leu323N	3.5
	S01	Tyr394OH	3.0
	N04	Leu323N	3.0
	N04	Leu323O	3.3
	N04	Phe322N	3.2
	N05	Phe322N	2.8
	Others:		
	N01	Leu323O	3.1
	N06	Leu323O	2.8
BPTES (3UO9)	Thiadiazol group:		
	SBA	Tyr394OH	3.3
	SAZ	Tyr394OH	3.3
	NAT	Phe322N	3.0
	NAV	Phe322N	3.1
	NAV	Leu323N	3.0
	NAU	Phe322N	2.9
	NAU	Leu323N	3.0
	NAS	Phe322N	3.1

(*Continued*)

Inhibitor	Inhibitor atom	Interacting cKGA atom	Distance (Å)
	Others:		
	OAA	Lys320NZ	3.5
	NAW	Leu323O	2.8
	NAX	Leu323O	3.0

Supplementary Table S2: Hydrophobic interactions for cKGA with inhibitors

Inhibitor	Inhibitor atom	Interacting cKGA atom	Distance (Å)
CB-839	C04	Leu321CD1	3.8
	C20	Tyr394CE1	3.9
	C20	Phe322CD2	4.0
	C23	Tyr394CE1	3.7
	C23	Phe322CD2	4.0
	C33	Glu325CG	3.9
1*S*, 3*S*-CBTBP	C04	Glu325CG	3.5
	C06	Lys322CD	3.3
	C06	Leu321CD2	3.4
	C12	Phe322CD2	3.7
	C12	Tyr394CE1	3.9
	C13	Phe322CE2	3.3
	C14	Tyr394CE1	3.9
	C22	Lys322CD	3.6
	C26	Glu325CG	3.4
BPTES (3UO9)	CAP	Phe322CE1	3.6
	CAO	Phe322CE1	3.7
	CAO	Tyr394CE2	4.0
	CAC	Glu325CG	3.4

Hydrophobic interactions were determined using Protein-Ligand Interaction Profiler{Salentin, 2015}
